# Every Saturday

**Published:** 1867-07

**Authors:** 


EVERY SATURDAY.—With the first number of July "Every Saturday," a journal devoted to the re-publication of current foreign literature, enters upon its fourth volume. The eminent success which has attended this weekly periodical, clearly demonstrates the estimation in which it is held by the reading public, and we feel confident that in every family in which it has been received it is highly prized. The publications of Messrs. Ticknor & Fields have acquired too high a reputation for their high style of typographical art and elegant appearance, to require any especial notice.

